# Avenanthramides and avenacosides as biomarkers of oat intake: a pharmacokinetic study of solid and liquid oat consumption under single and repeated dose conditions

**DOI:** 10.1186/s12937-025-01204-7

**Published:** 2025-09-09

**Authors:** Marina Armeni, Tim Cardilin, Rikard Fristedt, Therese Karlsson, Caroline Orfila Jenkins, Elise Nordin, Panpan Qin, Mats Jirstrand, Karsten Kristiansen, Otto Savolainen, Rikard Landberg

**Affiliations:** 1 Department of Life Sciences, Division of Food and Nutrition Science, Chalmers University of Technology, Gothenburg, 412 96 Sweden; 2 Chalmers Mass Spectrometry Infrastructure, Chalmers University of Technology, Gothenburg, 412 96 Sweden; 3 Department of Systems and Data Analysis, Fraunhofer- Chalmers Research Centre for Industrial Mathematics, Gothenburg, 412 88 Sweden; 4 Department of Internal Medicine and Clinical Nutrition, Institute of Medicine, Sahlgrenska Academy, University of Gothenburg, Gothenburg, 405 30 Sweden; 5 Oatly Global Science and Innovation Centre, Science Village, Rydbergs Torg 11, Lund, 224 84 Sweden; 6 Department of Biology, Laboratory of Integrative Medicine, University of Copenhagen, Copenhagen, 2100 Denmark

**Keywords:** Avenanthramides, Avenacosides, Prediction model, Biomarkers of oat intake, Pharmacokinetic parameter

## Abstract

**Background:**

Avenanthramides (AVAs) and Avenacosides (AVEs) are unique to oats (*Avena Sativa*) and may serve as biomarkers of oat intake. However, information regarding their validity as food intake biomarkers is missing. We aimed to investigate critical validation parameters such as half-lives, dose-response, matrix effects, relative bioavailability under single dose, and in relation to the abundance of *Feacalibacterium prausnitzii*, and under repeated dosing, to understand the potential applications of AVAs and AVEs as biomarkers of oat intake.

**Methods:**

Twenty-one healthy participants consumed two oat products (solid and liquid) in a non-blinded randomized crossover study for the pharmacokinetics (PK) assessment of multiple AVAs (2p, 2c,2f, 2fd and 2pd) and AVEs (A and B). At phase I, postprandial data were collected after a single dose of either product. At phase II, fasting sample was drawn after a 4-days repeated dose setup. The postprandial data were used in a compartmental PK model and the PK parameters were consequently utilized to predict individual plasma concentrations, which were compared with the data of the second phase of the study.

**Results:**

T_max_ values were shorter in liquid compared to solid form for AVAs (0.7–1.6 h and 1.1–2.3 h, respectively). In liquid, T_1/2_ were 1.3 h (AVA 2p and AVA 2fd), 3.2 h (AVA 2f, AVE A) and 2.5 h (AVA 2pd, AVE B). In solid form, T_1/2_ were shorter for AVAs (1.4–2.6 h) compared to AVEs (3.3–3.8 h). The normalized area under the curve (AUC_norm_) was greater for liquid than solid form for AVA2p, 2f and AVE-A [0.7–27 nM∙h (liquid), 0.4–20.1 (solid)] while for AVE-B AUC_norm_ were comparable [1.8 ± 0.2 nM∙h (liquid),2.1 ± 0.3 nM∙h (solid)]. A pharmakcokinetic prediction model described 75% of the experimental plasma-concentration data from phase II, with good agreement (bias: -0.145 nM).

**Conclusions:**

AVAs are promising candidates as compliance biomarkers of oat intake in intervention studies regardless of the tested food matrices. However, due to their short elimination half-lives, their applicability in nutritional epidemiology where long-term habitual intake is of main interest, seems restricted.

**Clinical trial number:**

This study was registered at clinicaltrials.gov with the clinical trial number: NCT05511077, on August 22nd, 2022.

**Supplementary Information:**

The online version contains supplementary material available at 10.1186/s12937-025-01204-7.

## Background

**Fig. 1:**
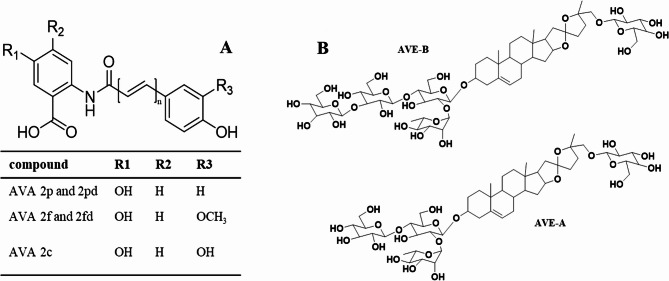
Chemical structures of AVA 2p 2pd, 2f, 2fd and 2c (**A**) and AVE A and AVE B (**B**). AVAs contain a cinnamic acid (right part of the structure) and an anthranilic acid moiety (left side of the structure). AVA 2pd and AVA 2fd contain one extra double bond (*n* = 2) compared to their counterpart avenanthramides. The R groups illustrate the position of potential substitution, and the specific substitution is presented in the table. The core of AVE structure is nuatigenin glycosylated at the C-26 with glucose and at C-3 with a trisaccharide for AVE A containing two glucose and one rhamnose unit, and with a tetrasaccharide for AVE B containing three glucose and one rhamnose unit

A diet rich in whole grain cereals has consistently been associated with lower risk of developing non-communicable diseases including type 2 diabetes, obesity, and cardiovascular disease [1–4]. Among the cereals, oats have gained specific attention owing to their unique nutrient composition [5], including mixed linkage beta-glucans with established cholesterol lowering and blood glucose dampening properties [6, 7], and unique bioactive compounds such as avenanthramides (AVAs) and avenacosides (AVEs). AVAs are phenolic compounds that contain a cinnamic acid and an anthranilic acid moiety **(**Fig. 1A). All their derivatives differ on the substitution of the R1-R3 functional groups and on the number of double bonds as presented in Fig. 1A. About 30 different avenanthramides have been reported in oats with AVA 2p, 2c and 2f (Fig. 1A) being the most prevalent and therefore the most studied [8, 9]. Avenacosides A and B are the main AVEs present in oat grains and leaves [10]. The core of their structure is nuatigenin glycosylated at the C-26 with glucose and at C-3 with a trisaccharide for AVE A containing two glucose and one rhamnose unit, and with a tetrasaccharide for AVE B containing three glucose and one rhamnose unit (Fig. 1B**)** [10].

Due to the unique presence of AVAs and AVEs in oats, they have been suggested as potential biomarkers of oat intake; however, information regarding their validity as food intake biomarkers (BFIs) is currently lacking [11]. Fundamental criteria of a BFI candidate are their specificity to particular foods (plausibility) and lack of matrix interference in diverse studies (robustness), their comparability to other dietary instruments (reliability), their absorption, distribution, metabolism and excretion (ADME) and the bioavailability together with pharmacokinetic (PK) properties such as half-life and dose response [12–15]. A recent work from Cuparencu et al. introduced the use of data-driven approaches as an alternative to correlation-based analyses for the evaluation of biomarkers of food intake (BFIs) with traditional food intake assessment methods. These strategies assess BFI reliability and differentiate BFIs based on their potential to predict food intake, while also mitigating biases associated with self-reported dietary registrations [14].

In vitro studies have shown that bioaccessibility of phenolic compounds from the gastrointestinal (GI) tract is partially depended on their interaction with other components of the food matrix, such as proteins and carbohydrates [16–19]. These interactions are affected by food processing, the matrix, and physicochemical properties of both the phenolic compounds and the interactive components [17, 20]. So far in vivo, only a few studies have studied the PK parameters of AVAs and the identification of their metabolites after acute consumption of oat-based products in humans [21–24]. Further, *Feacalibacterium prausnitzii* is known for its contribution to intestinal health, and preventive associations with metabolic perturbation and type 2 diabetes risk [25]. In the context of avenanthramide metabolism, one study correlated the abundance of *F. prausnitzii* to AVA metabotypes and specifically their ability to transform AVAs to their dihydro-counterparts [26]. Such metabotypes may exhibit different health benefits of consuming oats, which needs to be explored further in future studies.

Moreover, in an animal study the PK parameters of AVEs were studied in urine samples together with their gut microbiota metabolites [27]. With regards to AVEs, neither the impact of the food matrix on their bioavailability and their subsequent bioefficacy in vivo, nor their dose response in plasma has been thoroughly investigated.

The aim of this study is to assess AVAs and AVEs for their applicability in nutrition as biomarkers of oat intake. The approach was to evaluate the pharmacokinetics (PK) of the AVEs (AVE A and AVE B) and AVAs (AVA 2p, AVA 2c, AVA 2f, AVA 2pd and AVA 2fd) in humans after consumption of solid and liquid oat-based foods in a single and repeated dose setting. Then, PK models were established to best describe the ADME of each AVA and AVE and to simulate plasma concentrations of these compounds after different consumption doses and frequencies of consumption. The dose of AVAs and AVEs was extrapolated to doses of oat products and the experimental data of the repeated dose phase were used to assess the simulations and therefore the potential application of AVAs and AVEs as dietary tools. Also, as an exploratory analysis it was investigated if the relative bioavailability of AVAs was associated to *Feacalibacterium prausnitzii* abundance.

## Methods

### Study design

**Fig. 2:**
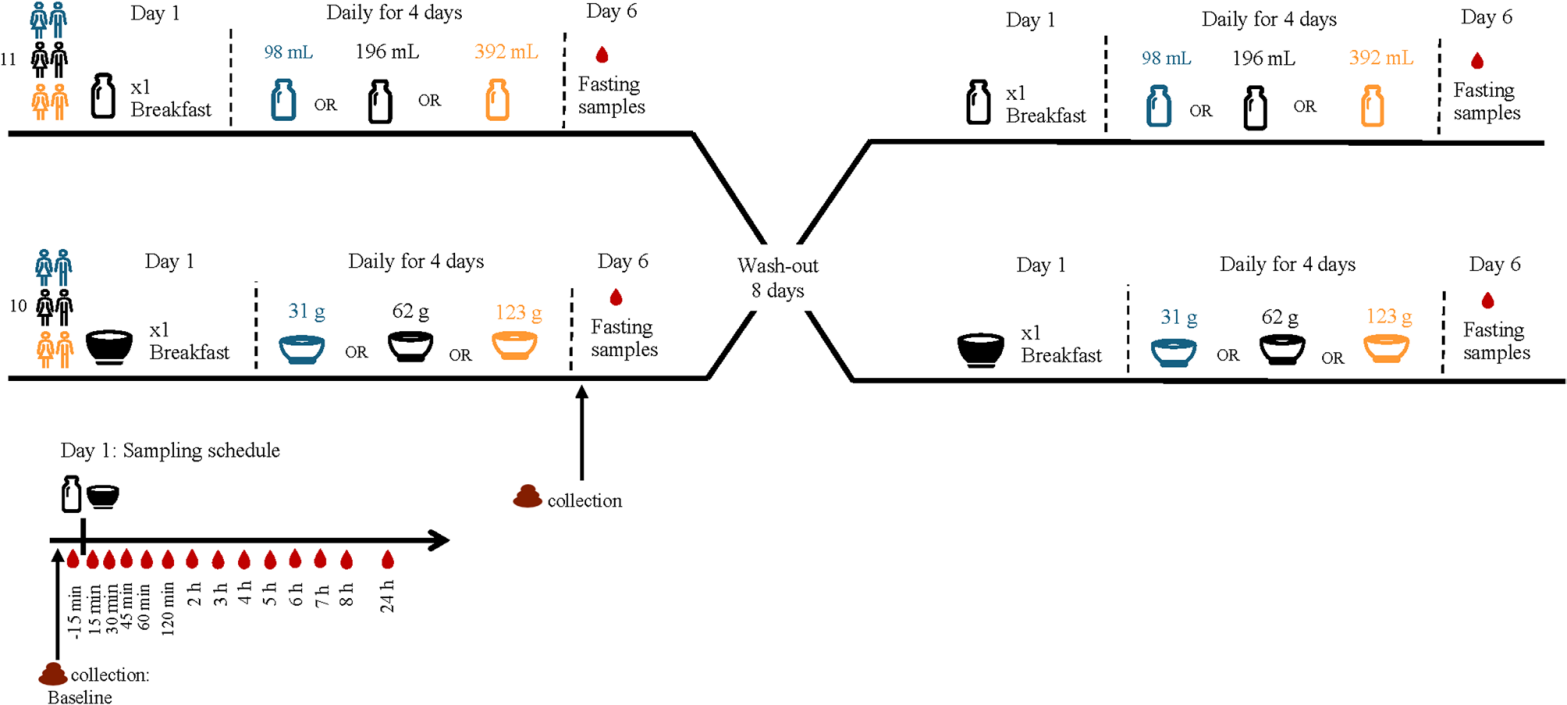
This study was conducted as a non-blinded, randomized crossover study where 11 men and 12 women consumed oat-based products in liquid and in solid form as single doses in the first phase of the study. In the second phase and after the single dose, the participants were randomized to receive one of three different doses of liquid or solid oats to consume three times per day for 4 days. On the fifth day of phase II a fasting sample was drawn. After a wash-out period, the participants repeated the same process with the product that they did not consume previously

A non-blinded, randomized two-way crossover study with two different oat products (solid or liquid) was designed to investigate the AVA and AVE PK after a single dose and after repeated dosing at three different doses, for respective food (Fig. 2). Since this is the first study of its kind, it is impossible to make any formal calculation of the number of subjects needed to show meaningful differences in plasma biomarker concentrations, since the variation is unknown. However, similar studies aimed for BFI discovery and validation of dose-response have typically used 5–30 subjects [28, 29]. We therefore considered 23 individuals as a good compromise between feasibility and possibilities to assess variation in biomarker concentrations, estimate pharmacokinetic parameters, dose response and performing modelling with good precision. A scientist not involved in the study, conducted the randomization using the RAR method (RandomizeR package) [30] in R software environment [31] and provided lists with allocated treatment sequences. The study was conducted in two phases. In the first phase, blood samples were drawn from 11 men and 12 women for 24 h after consumption of a single dose of either a solid or a liquid oat product on-site. Breakfast and lunch were also provided on-site. A single dose of solid (62 g of oat flakes) or liquid (196 mL of oat drink) containing individual AVAs and AVEs shown in Table 1 were consumed, respectively. Blood plasma samples were drawn for 24 h (0 h, 0.25 h, 0.5 h, 0.75 h, 1 h, 1.5 h, 2 h, 3 h, 4 h, 5 h, 6 h, 7 h, 8 h, 24 h). After the 24 h, at the second phase of the study, the corresponding product was further consumed three times per day for four days at either a low, medium, or high intake dose (98, 196 and 392 mL for liquid oats and 31, 62 and 123 g for oat flakes with corresponding contents of AVAs and AVEs (Table 1) in a Latin square design. Therefore, a total number of 8 participants (2 males and 6 females) were randomized to the low dose, 9 participants (3 males and 6 females) for medium dose and 6 participants (3males and 3 females) for the high dose. Participants consumed the provided products at home and were instructed to follow their habitual diets with restriction on the consumption of oat products. A list of relevant foods was provided as a guide. The oat flakes were pre-portioned into individual bags and could be consumed without heating, mixed with yoghurt, cottage cheese, sour milk or any liquid. Participants were instructed to consume the full amount, including the liquid or food the oats were mixed with. Liquid oat products were weighed prior consumption, and they were to be consumed without any additional food. In all cases the time of oat intake was recorded in a food journal. On the fifth day of phase II, a fasting sample was drawn. Fecal samples were collected at baseline and at the end of phase II of the intervention. Between each study period, there was a wash-out period of eight days where participants consumed their habitual diet. After the washout, participants started with a single dose and repeated dosing for the other product in the same way as for the first. The participants were instructed to avoid foods containing oats during the last three days prior to the start of the study and during the last three days of the wash-out period. Compliance was assessed based on information provided in a compliance journal during the study weeks as well as through 2-day food records. The oat products used in the study were provided to the participants. The flakes for the single dose were consumed with water ad lib and for the repeated dose they could be mixed with water, yoghurt, milk, sour milk or cottage cheese with the condition that all the amount of the oat product is consumed. The oat drink was consumed without any other product.

**Table 1:** Content of AVAs and AVEs in the different doses of liquid and solid oat product consumed in phase I and phase II of the intervention. The content is expressed per dry weight basis per portion and was measured after a freeze-drying process for the liquid product. The moisture of the oat drink was considered and calculated at 87% of the total weight. Measurement conducted with Mettler Toledo halogen moisture analyzer HE73 (230 V)

	**Single Dose [nmol and (mg)] **									
	**AVA ** **2p**	**AVA** ** 2c**	**AVA ** **2f**	**AVA ** **2pd**		**AVA 2fd**	**AVE** **A**	**AVE ** **B**	**AVA total**	**AVE total**
**Liquid** **(196 mL)**	902 (0.3)	647 (0.2)	1372 (0.5)	593 (0.2)		793 (0.3)	13644 (14.5)	2068 (2.5)	4307 (1.4)	15712 (17.0)
**Solid** **(62 g)**	10110 (3.0)	4883 (1.5)	7274 (2.4)	5260 (1.7)		3792 (1.4)	30804 (32.8)	3184 (4.0)	31320 (10.0)	33988 (36.7)
	**Low Dose (nmol and (mg))**									
	**AVA ** **2p**	**AVA ** **2c**	**AVA** ** 2f**	**AVA ** **2pd**	**AVA ** **2fd**		**AVE** ** A**	**AVE ** **B**	**AVA total**	**AVE total**
**Liquid** **(98 mL)**	451 (0.1)	323 (0.1)	686 (0.2)	297 (0.2)	396 (0.3)		6822 (7.3)	1034 (1.3)	2153 (0.7)	7856 (8.5)
**Solid** **(31 g)**	5055 (1.5)	2442 (0.8)	3637 (1.2)	2630 (0.9)	1896 (0.7)		15402 (16.4)	1592 (2.0)	15660 (5.0)	16994 (18.3)
	**Medium Dose (refer to Single Dose)**									
	**High Dose [nmol and (mg)]**									
	**AVA ** **2p**	**AVA ** **2c**	**AVA ** **2f**	**AVA 2pd**	**AVA 2fd**		**AVE ** **A**	**AVE ** **B**	**AVA total**	**AVE total**
**Liquid** **(392 mL)**	1803 (0.5)	1294 (0.4)	2744 (0.9)	1187 (0.4)	1586 (0.6)		27289 (29.0)	4136 (5.0)	8614 (2.8)	31424 (34.1)
**Solid** **(123 g)**	21618 (6.5)	10533 (3.3)	15875 (5.2)	11022 (3.6)	8378 (3.0)		69222 (73.6)	6816 (8.4)	67425 (21.6)	76038 (82.0)

### Participants

Twenty-three participants (11 men and 12 women) from the Gothenburg area, Sweden were enrolled according to the inclusion criteria of age (between 18 and 80 years), body mass index (BMI) (18.5–30.0 kg/m^2^), fasting glucose (≤ 6.1 mmol/L), low density lipoprotein (LDL) cholesterol (≤ 5.30 mmol/L) and triglycerides (≤ 2.60 mmol/L). Exclusion criteria included presence of food allergies or intolerances preventing the consumption of any products included in the study, difficulties to sufficiently understand written and spoken Swedish in order to provide written consent and understand the provided instructions and information, pregnancy, lactation or planning of pregnancy during the study period, antibiotic use for the last 3 months, blood donation or participation in a clinical trial with blood sampling within 30 days prior to screening visit and throughout the study, history of GI conditions possibly affecting absorption and metabolism (like ulcerative colitis, Crohn’s disease, etc.), previous major GI surgery (like Gastric Bypass, intestinal resection etc.), type I diabetes and thyroid disorder. All participants signed an informed written consent during the screening visit and before screening procedures started. A diagram on the enrollment process is available on supplementary Figure S1. This study was conducted according to the Declaration of Helsinki and approved by Swedish Ethical Review Authority.

### Sample collection and storage

All blood samples were collected into EDTA-coated tubes by a trained nurse. During the single dose day, the samples were drawn via a peripheral venous catheter and the participants were fasting for at least 8 h (e.g. 22:00 the night before). The blood tubes were centrifuged at 2500 x g for 10 min and at 4 °C directly after sampling and plasma then aliquoted on a cooling box and stored at −80 °C until analysis. Fecal samples were collected at home by the participants as they were instructed. The samples were kept in the home freezer until the next study visit, transported to the clinic in a cooling bag with frozen cooling blocks. To facilitate easier and convenient collection of samples, the participants were given a fecal kit collection (QIAamp fast DNA Stool mini kit, from QIAGEN N.V., Venlo, The Netherlands). Upon arrival to the clinic, fecal samples were initially stored at − 20 °C for up to 7 days, before being transferred to a − 80 °C freezer for long-term storage.

### Food products and chemicals

The solid food products (malted oat flakes; SPC flakes) [32] used were purchased from a local pharmacy store and the commercial oat drink (10% oats) was provided by Oatly AB (Malmö, Sweden). NaH_2_PO_4_ was from Supelco (Bellefonte, Pennsylvania, USA), ascorbic acid was purchased by Fluka Chemie GmbH (Buchs, Switzerland) and from Sigma Aldrich (Saint Louis, Missouri, USA). Ethylenediaminetetraacetic acid (EDTA), formic acid, AVA 2p, AVA 2f, AVA 2c, Tranilast^®^, ginsenoside Rb1 and β-glucuronidase/sulfatase (type H-2) from *Helix pomatia* were from Signa Aldrich (Saint Louis, Missouri, USA). The solvents of methanol and acetonitrile (CHROMASOLV™) were from HoneyWell (Charlotte, North Carolina, USA). Avenacoside A and B standards were provided by Professor Shengmin Sang, University of North Carolina. Standards of AVA 2pd and AVA 2fd were provided by Professor Lena Dimberg, Swedish University of Agricultural Sciences. The solid phase extraction (SPE) plates (Oasis HBL uElution Plate 30 μm were Waters™ (Milford, Massachusetts, USA). The sample analysis was completed using a Exion UHPLC coupled to QTRAP 6500 + from ABSciex (Redwood City, CA, USA) and the analytical column (Inetrsil ODS-3 3 μm, 3 × 150 mm) was from Fisher Scientific International Inc.

### Analysis of AVA and AVE in plasma

The samples were analyzed in a blocked randomized fashion including plasma samples from the same subject after consumption of both oat products in the same block. The extraction of total AVAs and AVEs was adapted from the original extraction method [24] with modifications. The modifications were focused on optimizing the time for enzymatic cleavage of glucuronide and sulfate conjugates and on the improvement to ensure quantitative extraction recoveries of avenacosides. Briefly, 200 uL of plasma with 20 uL of 0.4 M NaH_2_PO_4_ solution containing ascorbic acid and EDTA (200 and 1 mg/mL) and internal standards (ISs) (6.7nmol/L and 0.7 nmol/L for Ginsenoside Rb1 and Tranilast^®^, respectively), were placed on an equilibrated HBL uElution SPE plate and incubated with 20 uL of glucuronidases/sulfatases (98/2.4 units/L) for 2 h and at 37 °C. After incubation the analytes were extracted with 1 mL acetonitrile and purified with SPE. The extractants were dried and reconstituted with 100 uL of 1:4 v/v, acetonitrile: milliQ water containing 0.1% formic acid and vortexed for 10 min before transferring to vials. The vials were centrifuged for 10 min prior analysis. The extraction recovery was calculated with spiked samples in triplicates.

### LC-MS/MS methods

Prior to the intervention study, the concentrations of AVAs and AVEs in the consumed products were measured with a method previously developed in our laboratory [33]. Samples were analyzed by injecting 8 *µ*L into a Exion LC (Shimadzu, Kyoto, Japan) coupled to a QTRAP 6500+ (AB Sciex). The analytes were separated on a reversed phase column (inertsil ODS-3; 3 μm, 3 × 150 mm) by using gradient elution of milliQ water with 0.1% formic acid (mobile phase A) and acetonitrile with 0.1% formic acid (mobile phase B). The gradient elution program was applied by increasing the percentage of mobile phase B from 0 to 15 within the first 3 min. The next 5 min mobile phase B rose to 65% and remained stable for 3 min. In the next half a minute, mobile phase B reached 100% and stayed in this level for another half a minute before re-equilibration. AVAs and Tranilast^®^ were measured in positive electrospray mode with retention times 7.8 min for AVA 2p, 7.3 min for AVA 2c, 8.1 min for AVA 2f, and for Tranilast^®^ at 10.3 min (Figure S2A). AVA 2pd and AVA 2fd eluted at 8.4 and at 8.3 min. Respective elution times for AVEs and Ginsenoside Rb1 in negative electrospray mode were measured at 7.0, 6.8 and 7.3 min for AVE A, AVE B and Ginsenoside Rb1, respectively (Figure S2B). The curtain gas (CUR) was at 35 psi, collision gas (CAD) at 10 V and the ion spray voltage at ± 4500 V. The temperature was set at 500 °C and gas one (GS1) and two (GS2) at 60 psi. Multiple reaction monitoring (MRM) transitions for AVAs and their IS were registered in positive electrospray mode and they were 299.9◊146.9 and 299.9◊119.1 for AVA 2p, 329.9◊176.9 and 329.9◊145.0 for AVA 2f, 315.9◊162.9 and 315.9◊145 for AVA 2c, 356.07◊203.1 and 356.07◊175.1 for AVA 2fd, 326.0◊172.950 for AVA 2pd and Tranilast^®^ used as IS was registered at 328◊190.9 and 328◊163. In negative mode the transitions for AVE A were 1061.4◊59.00 and 1061.4◊71.00, for AVE B were 1223.5◊59.00 and 1223.5◊899.00 and for ginsenoside Rb1 were 1107.4◊789.1 and 1107.4◊58.9. All MRMs were optimized with the use of standards. All the data were integrated by using MultiQuant (ABSciex). The concentrations of the analytes were calculated based on calibration curves of standards for AVA 2p, AVA 2f, AVA 2c and for AVE A and AVE B and the lower limits of quantification (LLOQs) and of detection (LLOD) were determined by LINEST function in Microsoft excel. The calibration range was from 0.2 to 20 ppb for AVAs and 2–40 ppb for AVEs. The concentrations of AVA 2pd and AVA 2fd were estimated using calibration curves of AVA 2p and AVA 2f, respectively.

### Pharmacokinetic modelling and statistics

A compartmental model with two absorption compartments and a plasma compartment with linear elimination for each molecule separately described better the experimental data from the single dose intake compared to a compartmental model of a single absorption compartment (Figure S3). Parameter estimation was performed using the non-linear mixed-effects approach with lognormal inter-individual variability in all three model parameters: distribution volume over bioavailability ($$\:V/F$$), absorption rate ($$\:{k}_{a}$$), elimination rate ($$\:{k}_{e}$$), (R package nlmixr2 [34]). The results were reported as population means with coefficient of variation (Table S1). The area under the curve (AUC) and Cmax were normalized (AUCnorm) for the nmol intake of each molecule. Statistical analysis was conducted with R software environment [31]. The compounds were compared for their PK parameters within the same product and between products using repeated ANOVA approach (lme4 package [35]) and then by pairwise comparison adjusted for post hoc Tukey-Kramer honestly significant difference. All statistical analyses were performed on log- transformed data. Based on the PK models built on the single-dose data, a linear relationship ($$\:Dose=C\bullet\:{\raisebox{1ex}{$V$}\!\left/\:\!\raisebox{-1ex}{$F$}\right.\bullet\:k}_{e}\bullet\:\varDelta\:t)$$ was derived between dose and dosing interval (intake frequency, $$\:\varDelta\:t$$) to estimate the AVA intake needed to reach an average concentration (at steady state) that is measurable above lower limit of quantification (LLOQ). To translate the intake of AVAs from the linear relationship (*Dose*) to corresponding oat flake product intake, we estimated the content of AVAs in different products using reported data from the literature and assigned values, where possible. For the flakes, information in literature was available for AVA 2c, AVA 2p and AVA 2 f. Therefore, an average value for these compounds per food product was assigned [33, 36] while for the oat drink and for the less known AVAs; AVA 2pd and AVA 2fd, an average value based on the measured products in our laboratory was used (Table S2). In the second phase of the intervention, following a repeated dosing period of 4 days, plasma concentrations were determined over a dosing interval recorded by the individuals and were compared to their corresponding predicted values in blood. This comparison allowed for an assessment of the model’s accuracy in predicting AVAs’ behavior under repeated dosing conditions. Only experimental data above the limit of quantification were used for the comparison. Further, the data were tested for outliers with the interquartile range (IQR) approach and five observations were considered outliers and were removed.

### Fecal sample preparation for metagenomic analysis

DNA was extracted from approximately 35 mg of each of the 451 fecal samples using the NucleoSpin Soil kit (Macherey-Nagel, 740780.250 M), following the manufacturer’s instructions. DNA yield was quantified using the Qubit 1X dsDNA Broad Range assay kit (Thermo Fisher Scientific, Q33266). For metagenomic shotgun sequencing, libraries were prepared with the MGIEasy Fast FS DNA Library Prep Set (MGI Tech Co., Ltd., 940-000030-00, Werheim, Germany), using 400 ng of DNA per sample. The library preparation process included DNA shearing for 12.5 min, followed by end repair, A-tailing, and magnetic bead-based cleanup. Adapter ligation and cleanup were then performed, followed by PCR amplification and magnetic bead-based size selection of the final library product. Library quality was assessed using the Qubit 1X dsDNA HS Assay Kit (Thermo Fisher Scientific, Q33231) and the Agilent High Sensitivity D1000 Assay Kit (Agilent Technologies, 5067–5584 and 5067–5585, Santa Clara, CA, USA). Pooled libraries were circularized, barcoded, and used as templates for DNA nanoball preparation. The nanoballs were then analyzed on the DNBSEQ-T7 platform (MGI Tech Co., Ltd.) using the DNBSEQ-T7RS High-throughput Sequencing Set (FCL PE150), according to the manufacturer’s guidelines.

### Metagenomic data processing

Raw metagenomic reads underwent quality control to remove low-quality sequences, adapters, and contaminants using Fastp [37] (version 0.23.2, default parameters). High-quality reads were filtered to exclude human reads (GRCh38_noalt_as) using Bowtie2 [38] (version 2.5.3). The quality-filtered reads were then aligned to the taxonomy database using MetaPhlAn (version 4.0.6, default parameters) [39] to generate relative abundance profiles. For inclusion in the analysis, a species was required to be present in at least three samples.

### Data analysis of gut microbiota

For the seven metabolites AVE A, AVE B, AVA 2c, AVA 2f, AVA 2fd, AVA 2p, AVA 2pd analysed at phase I after the single dose of oat products, C_max_ and area under the curve (AUC) were stratified based on less than the median or more or equal to the mean. Thereafter, for each metabolite, it was investigated whether the relative abundance of *F. prausnitzii* at baseline could predict the strata since this bacterium has previously been reported to be responsible for production of their dihydro-metabolites [26]. Logistic regression with a binomial distribution and a logistic link function was used to separately analyze the liquid and solid products. When analyzing liquid and solid products together, a generalized linear mixed model with a binomial distribution and a logistic link function was used, with identity included as a random factor.

## Results

### AVA and AVE content in consumed products

**Fig. 3:**
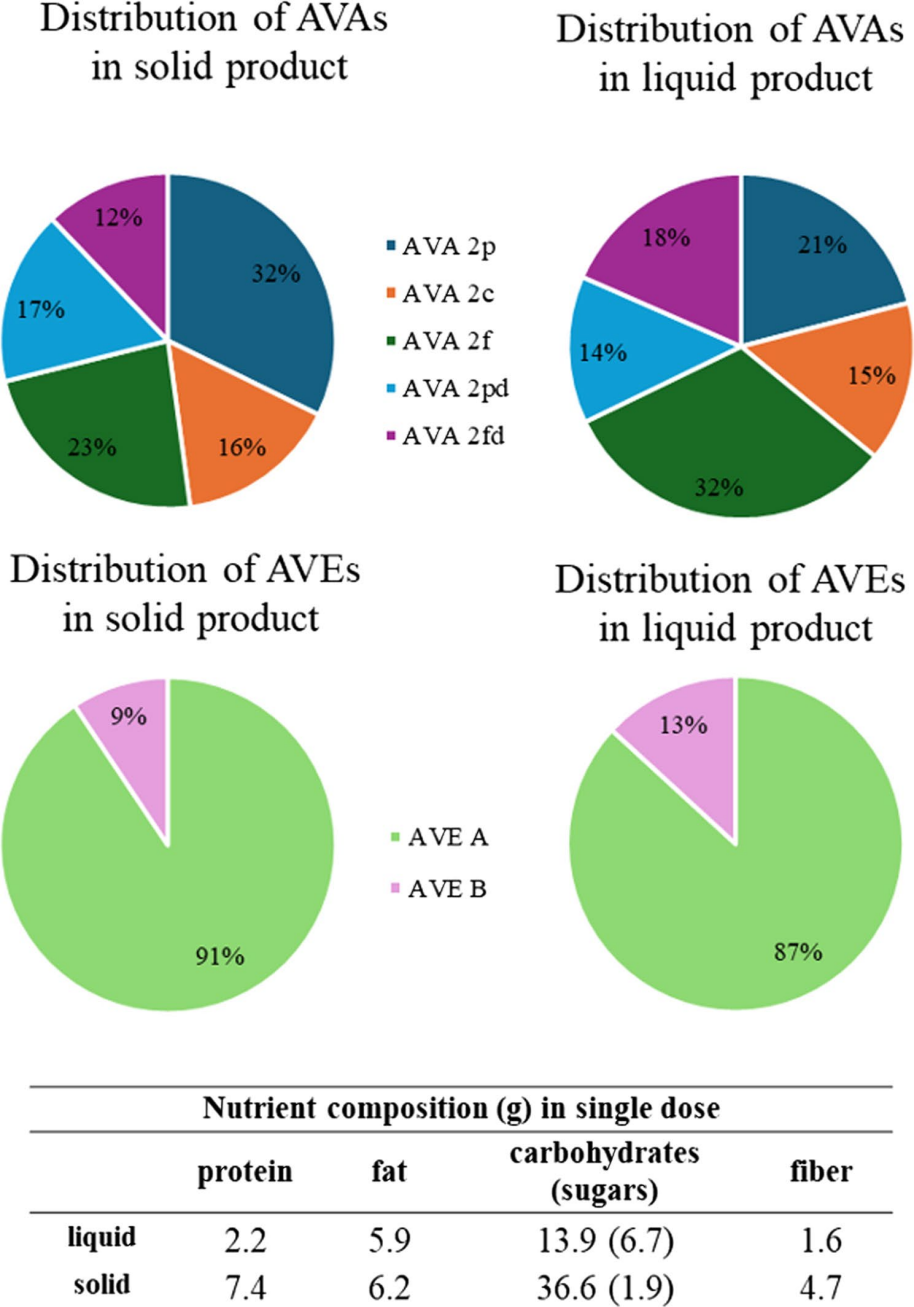
Composition of AVAs and AVEs in the products, measured in our laboratory. The contents in the upper pie charts in percentage the content of measured AVAs. The lower pie charts, respectively, present the content of AVEs. The left side illustrates the composition in oat flakes and the right-side composition in oat drink. The nutrient composition table corresponds to the amount of single dose, 62 g for the solid and 196 mL for the liquid product. In the nutrient composition table carbohydrates correspond to carbohydrate content excluding fiber and sugars are presented in brackets as part of carbohydrate content

The distribution of AVAs and AVEs did not differ between the tested products (Fig. 3). In the liquid product AVEs were 4 times higher compared to AVAs while in the solid product they were present in equal amounts. The total amount of AVAs and AVEs was 7- and 2-times higher, respectively, in the solid than in the liquid product for each dose (Table 1). The selection of the food material was based on the AVA content, as AVA/AVE ratios across formulations were not similar. Additionally, the nutrient composition of the two products was similar with regards to the lipid content whereas dietary fiber, proteins and carbohydrates were lower in the liquid product (Fig. 3).

### Participants

Of 38 assessed participants, 15 were excluded due to unmet inclusion criteria (*n* = 4), nonparticipation (*n* = 3) or other reasons (*n* = 8). Of the 23 randomized participants two dropped out and did not complete the intervention due to sickness at the second period of the crossover study (*n* = 1) and due to difficulties with venous blood sampling (*n* = 1) (Figure S1). Of the 21 participants that completed the study and were included in the analysis, 9 were men and 12 were women. The average BMI was 23.8(± 2.4) kg/m^2^ and the age was 49 (± 19) years. All participants who completed the study were considered compliant (> 80% of intervention foods were consumed).

### LC-MS/MS analysis of AVAs and AVEs in plasma

The recovery of the method was 71–83% and the upper limit of the linear range was 60 nmol/L for AVAs and 38 nmol/L for AVEs. The LLODs and LLOQs were later used for the estimation of minimal intake dose and frequency of oat products to achieve measurable plasma concentrations, i.e. the lowest possible intake dose and frequency allowing reliable quantification (Table S3).

### Pharmacokinetics of AVAs and AVEs

**Fig. 4:**
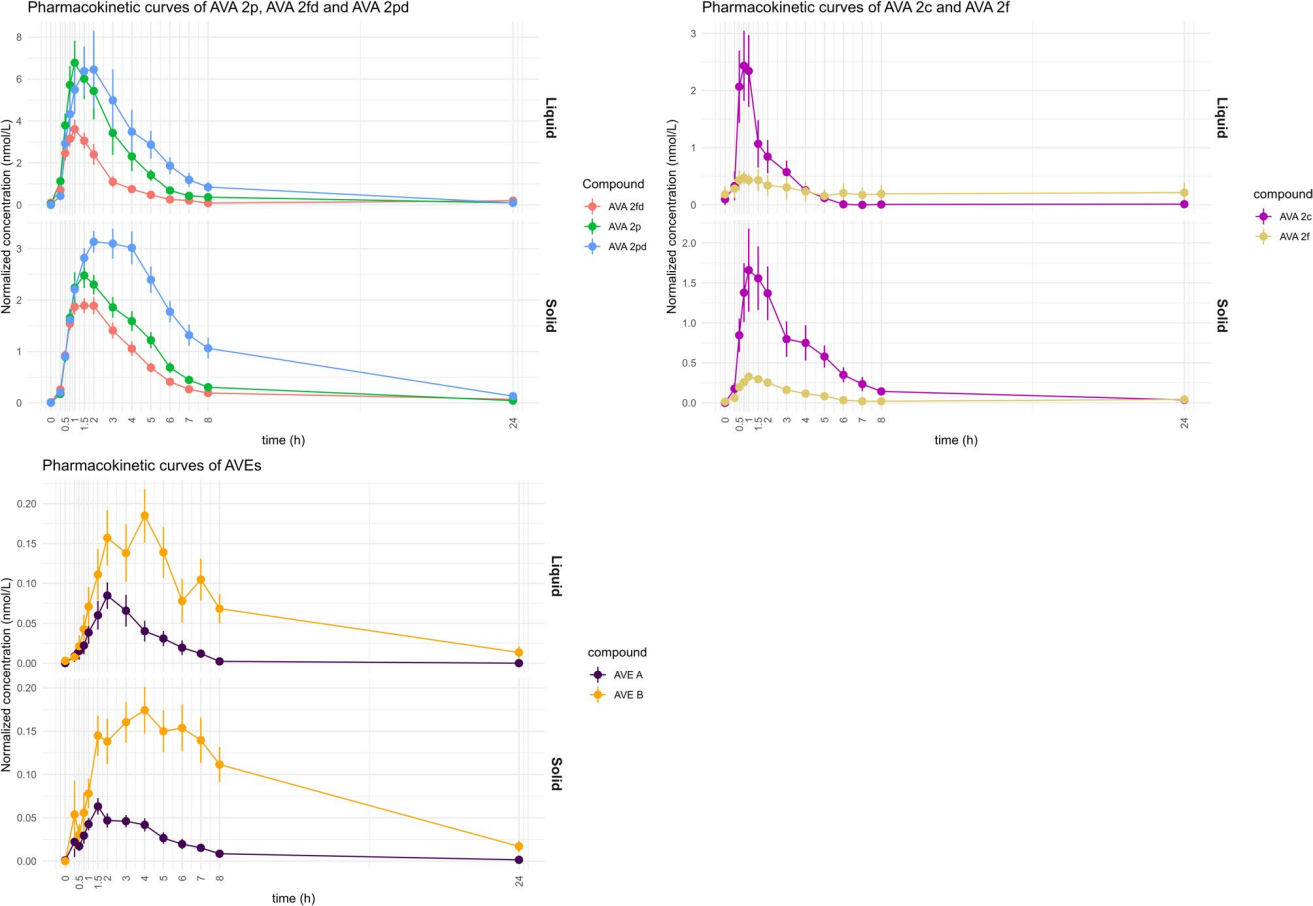
PK curves of AVAs and AVEs after single dose of oat products in liquid or solid forms. The data points correspond to the average values of the concentrations at the given time point from all participants, together with the standard error bars

Plasma concentrations of all seven analyzed molecules after consumption of liquid and solid product were well described by a simple linear compartment model with two absorption states for each individual molecule (Figure S3). The profile of the PK curves is illustrated in Fig. 4 and the PK parameters of AVAs and AVEs in the liquid and solid oats were derived from the PK model (Table 2). The time at which the concentration was at maximum level (T_max_) ranged from 0.7 to 1.6 h and from 2.5 to 3.0 h in liquid form for AVAs and AVEs, respectively, and from 1.1 to 2.3 h (AVAs) and 1.8–3.3 h (AVEs) in solid form. In general, T_max_ was significantly longer after consuming the solid matrix for all AVAs while the opposite was noted for AVE (A) Cmax was lower in the liquid form, from 0.7 to 4.9 nM for AVAs and 0.4 and 1.3 nM for AVEs compared to solid form (2.1–20.0 nM and 0.6-2.0 nM, for AVAs and AVEs, respectively). In contrast, when the maximum concentration was normalized for dose (C_max(norm)_), C_max(norm)_ was higher after consuming oats in liquid form compared to solid form for all compounds except for AVE (B) After consuming the liquid form, the half-life T_1/2_ of AVAs varied from 1.3 to 3.2 h and at 2.6 and 3.2 h for AVEs and the T_1/2_ in the solid form was 1.4–2.6 h and 3.3–3.8 h, for AVAs and AVEs respectively. Finally, the area under the curve normalized for dose (AUC_norm_) was calculated at 2.8–27.0 nM∙h for AVAs and at 0.7–1.8 nM∙h for AVEs in the liquid form and in the solid form at 1.3–20.1 nM∙h for AVAs and 0.4–2.1 nM∙h for AVEs. In general, even though AUCnorm followed the same trend as C_max(norm)_, only AVA 2p, AVA 2f and AVE A exhibit significant differences between the two matrices.

**Table 2:** PK parameters derived from the PK-model and compared between the two oat products with post-hoc Tukey significance test

T_max_ (h)							
oat form	AVA 2p	AVA 2c	AVA 2f	AVA 2pd	AVA 2fd	AVE A	AVE B
**Liquid**	1.21 ± 0.05^1^	0.73 ± 0.03^1^	0.98 ± 0.04	1.60 ± 0.07^1^	1.01 ± 0.04^1^	2.51 ± 0.11^1^	2.98 ± 0.13
**Solid**	1.91 ± 0.08	1.37 ± 0.06	1.11 ± 0.05	2.31 ± 0.10	1.53 ± 0.07	1.78 ± 0.08	3.29 ± 0.14
**C**_**max(norm)**_ **(nM)**							
** Liquid**	5.49 ± 0.56^1^	1.79 ± 0.18^1^	0.50 ± 0.05^1^	5.23 ± 0.53^2^	3.11 ± 0.32^2^	0.1 ± 0.01^2^	0.2 ± 0.02
** Solid**	1.98 ± 0.21	1.01 ± 0.11	0.29 ± 0.03	3.20 ± 0.34	1.94 ± 0.21	0.07 ± 0.01	0.2 ± 0.02
**C**_**max**_ **(nM)**							
** Liquid**	4.94 ± 0.50^1^	1.16 ± 0.12^1^	0.69 ± 0.07^1^	3.10 ± 0.31^1^	2.46 ± 0.25^1^	1.31 ± 0.14^2^	0.44 ± 0.05^2^
** Solid**	20.00 ± 2.12	4.91 ± 0.52	2.10 ± 0.22	16.84 ± 1.78	7.34 ± 0.78	2.03 ± 0.22	0.64 ± 0.07
**T**_**1/2**_ **(h)**							
** Liquid**	1.33 ± 0.10	1.58 ± 0.12^2^	3.19 ± 0.25^2^	2.45 ± 0.19	1.33 ± 0.10	3.17 ± 0.24	2.56 ± 0.20^2^
** Solid**	1.42 ± 0.11	2.13 ± 0.16	2.28 ± 0.18	2.63 ± 0.20	1.65 ± 0.13	3.32 ± 0.26	3.76 ± 0.29
**AUC**_**(norm)**_ (nM*h)							
** Liquid**	17.33 ± 2.19^1^	5.31 ± 0.67	2.79 ± 0.37^1^	27.01 ± 3.41	9.06 ± 1.14	0.68 ± 0.09^2^	1.77 ± 0.23
** Solid**	8.18 ± 1.07	4.42 ± 0.58	1.28 ± 0.17	20.10 ± 2.63	7.59 ± 1.00	0.43 ± 0.05	2.08 ± 0.27
**k**_**a**_ **(1/h)**							
** Liquid**	2.8 ± 0.2^1^	6.0 ± 0.3^1^	5.0 ± 0.3^2^	2.40 ± 0.1^1^	3.6 ± 0.2^1^	1.4 ± 0.1^1^	0.41 ± 0.02
** Solid**	1.50 ± 0.08	2.8 ± 0.16	3.8 ± 0.21	1.48 ± 0.10	2.2 ± 0.12	2.3 ± 0.13	0.46 ± 0.03
**k**_**e**_ **(1/h)**							
** Liquid**	0.52 ± 0.03	0.44 ± 0.03^2^	0.22 ± 0.02^2^	0.28 ± 0.02	0.52 ± 0.04^2^	0.23 ± 0.02	0.27 ± 0.02^2^
** Solid**	0.49 ± 0.04	0.33 ± 0.03	0.30 ± 0.02	0.26 ± 0.02	0.42 ± 0.03	0.21 ± 0.02	0.18 ± 0.03
**V/F**							
** Liquid**	111 ± 11^1^	430 ± 42^2^	1641 ± 168^2^	131 ± 13^2^	211 ± 21^2^	6718 ± 686^2^	2079 ± 212
** Solid**	250 ± 26	698 ± 71	2561 ± 262	189 ± 19	314 ± 32	11,055 ± 1130	2606 ± 266

^1^indicate*s* a significant level of difference *p*≤0.001, ^2^*p*<0.01 and ^3^*p*<0.05 for the same molecule between the two oat products in all the graphs. Significant difference among molecules for the same oat product is not noted

**Fig. 5:**
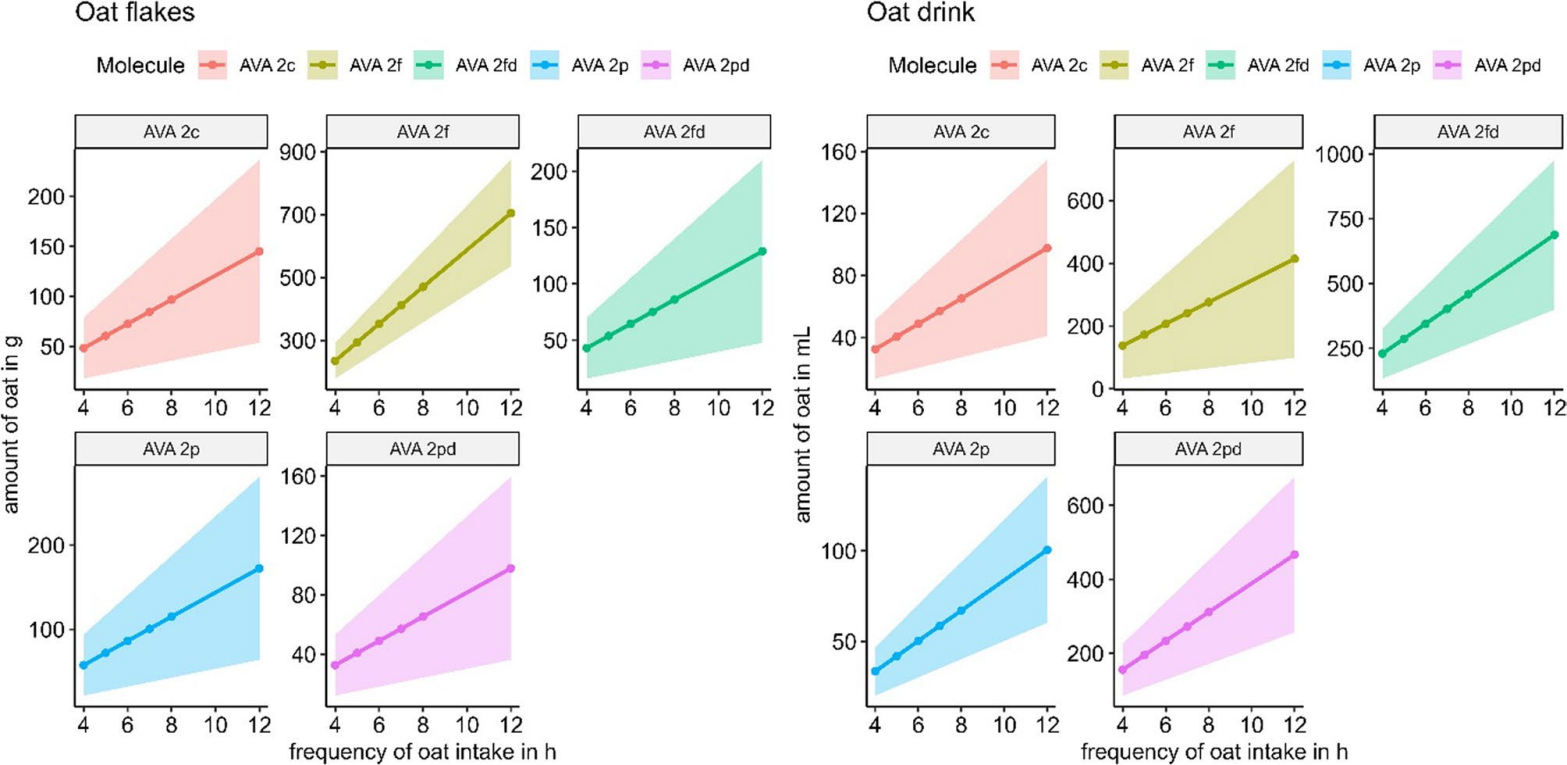
Relationship between estimated consumed amount of oat flakes (left side) and oat drink (right side) and frequency of consumption

The dose of an oat product needed to reach quantifiable plasma concentrations at different time intervals based on estimations for each individual AVA separately (Fig. 5). This estimation was not applied to AVEs due to the low bioavailability they exhibited in relation to their LLOQ levels of the method. For AVA 2c, 2p, 2pd and 2fd the average amount of oat flakes needed was estimated at about 30–60 g and for AVA 2f at 235 g if it would be consumed every 4 h per day. If it were consumed every 8 h, the amounts needed would increase to 65–115 g for AVA 2c, 2p, 2pd and 2fd and 470 g for AVA 2f to reach measurable levels in blood. For the liquid oats and for a consumption interval every 4 h, the estimated volume of approximately 30 mL would result in plasma concentration of above LLOQ for AVA 2p and 2c. For the same conditions the estimated volume for AVA 2f, 2pd and 2fd was between 140 and 230 mL. For intakes every 8 h, the corresponding estimated intake volumes needed were 65 mL for AVA 2p and 2c and at 280–460 mL for AVA 2f, 2pd and 2fd.

**Fig. 6:**
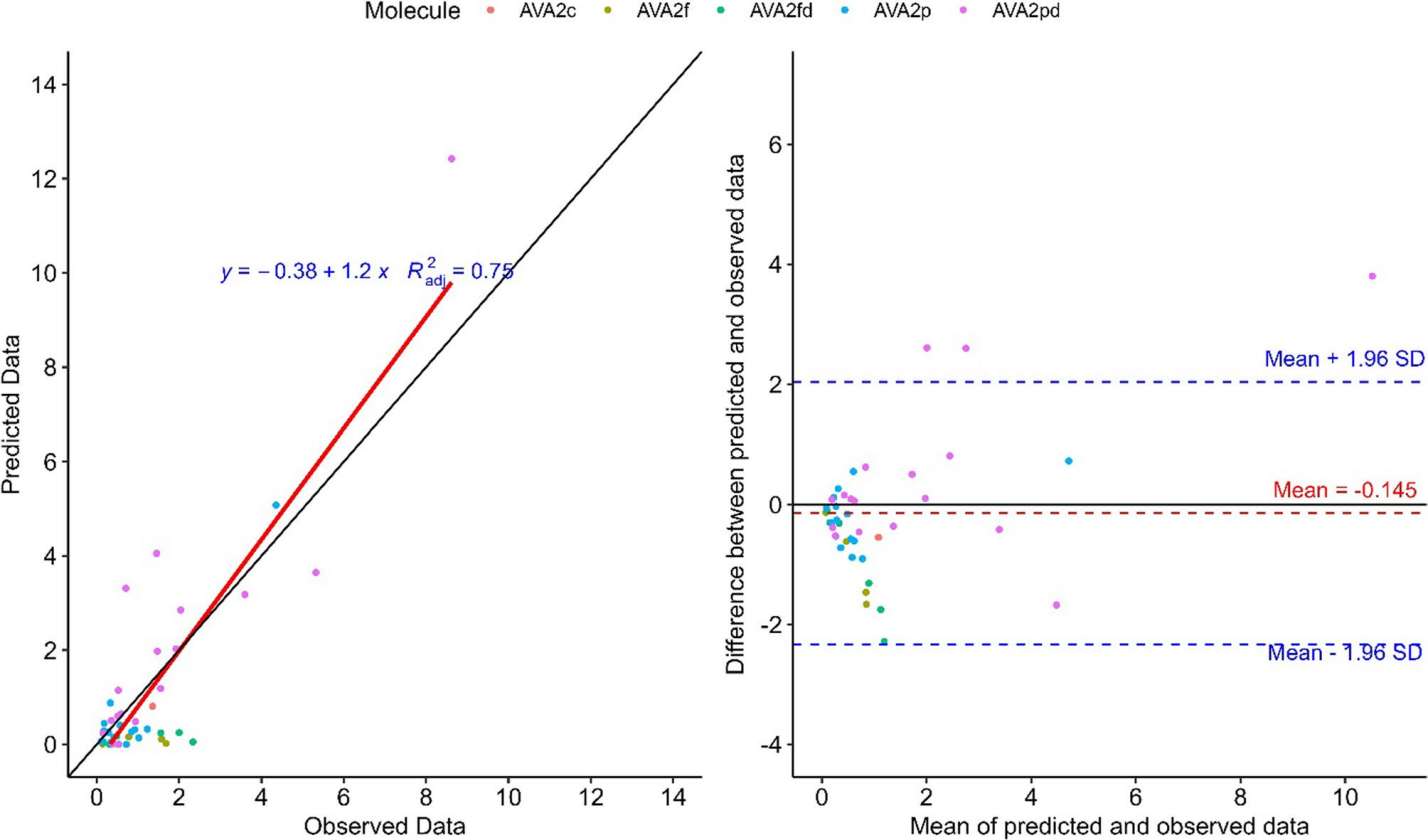
Goodness-of-fit plot (left graph) of predicted plasma concentrations and experimental data from the second phase of the intervention. Blant-Altman plot (right graph) for the predicted blood plasma concentrations against the average experimental blood plasma concentrations and the same dose intervals. The red dotted line indicates the mean difference between the predicted and the observed values and the blue dotted lines the 95% upper and lower limits of agreement. The total number of observations was 43

Goodness-of-fit plots were applied to assess the performance of the individual PK models in predicting AVA concentrations in the day 5 samples from the second phase of the intervention (Fig. 6). The model described 75% of the variation of the experimental data and the mean difference between the predicted and observed data was illustrated in the Bland-Altman plot with a minor bias at −0.145 nM when comparing predicted – measured values. Additional metrics were calculated and found at 0.32 nM for the the Root Mean Square Error (RMSE) and at 0.261 nM for the Mean Absolute Error (MAE).

### Determination of Faecalibacterium Prausnitzii

There were no significant associations observed between *F.prausnitzii* abundance at baseline and the Cmax and AUC of AVE A, AVE B, AVA 2c, AVA 2f, AVA 2fd, AVA 2p, AVA 2pd, regardless of whether the oat products were analyzed as liquid, solid, or combined.

## Discussion

Food intake biomarker candidates need to be validated and assessed for their application before their use in interventions or nutritional epidemiological studies. The data and the derived parameters from PK studies not only provide insights for the biomarker bioavailability, the time- and dose-responses but also, they assist on the utility of the biomarker by predicting doses and dose frequencies above which the biomarker is measurable. Even if a biomarker candidate may have a short elimination half-life and not being suitable to reflect long-term intake in epidemiological studies, they may be used as compliance markers in dietary interventions where the food they reflect is consumed regularly [14, 15].

This study investigated the PK parameters of AVAs and AVEs after intake of oats in two different food forms using volumes and amounts that align with typical daily consumption, such as a bowl of oat porridge or a glass of oat drink. Although our intention was to directly compare AVAs and AVEs between matrices, this was not possible, as equal amounts of AVE and AVA content in the two formulations did not represent commonly consumed amounts of the products. Rather, the aim was to characterize the individual pharmacokinetic behavior of each product under realistic consumption conditions, using commercially relevant formats. This approach enhances the practical relevance of the findings. Further, it utilized the PK information to estimate under which circumstances AVAs are quantifiable in blood circulation. The estimated results were compared with experimental data of repeated consumption to evaluate the consistency of the two data sets. To the best of our knowledge, this is the first study to utilize PK data to estimate under which intake conditions biomarkers of food intake can be utilized in nutritional studies.

After testing two compartmental PK models, with a single absorption compartment and with two absorption compartments, for data fitting, the second model was selected based on performance. The selection of the model with two absorption compartments is in line with the results on the PK curves where a shoulder peak appears at 3–4 h, indicating a secondary phase of absorption or a re-absorption due to enterohepatic circulation (Fig. 4). Thanks to evidence of polyphenol absorption in both the small and the large intestine, we believe that the shoulder effect is rather a result of a secondary absorption phase [40]. Further, the enhanced intensity of this effect in the solid compared to the liquid form could be explained by the expected larger interactions of AVAs with starch since the latter is present in higher amount in the solid compared to the liquid form (Table 2; Fig. 3). C_max_ increased with increasing dose for all compounds, but this relationship was not linear (Table 2; Fig. 3), implying that the food matrix plays a role in the absorption of AVAs. When C_max_ was normalized against the dose, it was higher after intake of oats in the liquid form than after intake of the solid product for all AVAs and AVE A suggesting higher relative bioavailability when consumed as liquid compared to solid form. A similar trend was observed for AUC, for AVA 2p, AVA 2f and AVE A where the AUC was significantly higher in the liquid compared to the solid form. The lower relative bioavailability of AVAs in the solid compared to the liquid oats may also be attributed to the higher presence of starch in the flakes (Fig. 3). Pharmacokinetic parameters were comparable with previous studies where they were estimated from urine or plasma for AVAs [22–24]. The variations in T_max_, T_1/2_, k_a_ and k_e_ among compounds within the same product suggested that absorption and elimination rates were dependent on the structure of the individual compound.

A recent in vitro study has shown that the bioaccessibility of AVA 2c increased in the presence of starch whereas of AVA 2p and AVA 2f decreased [41]. This is in line with the results in our study showing similar relative bioavailability of AVA 2c but different for AVA 2p and AVA 2f when comparing liquid vs. solid formulations. Moreover, protein-phenolic interactions have various effects on the bioaccessibility of phenolic compounds [17]. AVAs were studied recently in vitro for their bioaccessibility in the presence of milk proteins and the results showed a decreased bioaccessibility in the presence of casein and milk protein while it increased with whey protein [42]. In the control samples (digestion without proteins), the bioaccessibility of AVA 2c was lower by 4.6 and 3.9 times compared to AVA 2p and AVA 2 f. However, in the presence of whey protein it increased by 3.6 times while AVA 2p and AVA 2f exhibited a slight increase [42]. Understanding the impact of oat proteins on their interaction with AVAs can shed further light on the diversity of AVA bioavailability.

Moreover, polyphenols are extensively metabolized by gut microbiota [43] and regarding AVAs, the abundance of *F. prausnitzii* was previously found to be responsible for the conversion of AVAs to their dihydro-metabolites (DH-AVAs) [26]. In our analysis no significant differences were observed between Cmax and AUC for AVAs and the relative abundance of *F. prausnitzii* at baseline after correction for multiple testing. AVAs were converted to DH-AVAs solely by gut microbiota and the earliest conversion reported at 4–6 h time interval [26] Therefore considering the short T_1/2_ of AVAs, this conversion is reflected in plasma after 2–3 h of ingestion, at the second half of the PK curve, and its contribution to the AUC could be masked by the total inter-individual variability of AUC. Thus, the associations between AVAs and *F. prausnitzii* abundance might be hidden.

There is only one study that investigated the PK parameters of AVEs in urine [27]. The T_max_ of excretion rate from urine data of that study does not overlap with the expected T_max_ for excretion based on plasma-data in our study, but renal clearance of AVEs was less than 3% [27]. This finding indicates that another path than renal clearance might be their main elimination path and/or that their absorption and bioavailability is low. Similarly, the low relative bioavailabilities we observed suggest that AVEs are poorly absorbed. Further their logP_o/w_ values, as calculated by SwissADME [44] (−1.64 and − 3.26, for AVE A and AVE B respectively), suggest high hydrophilicity and, due to their large size, a low likelihood of absorption in the GI tract. Consequently, AVEs were not included in the dosing prediction modelling.

A prediction model was utilized to simulate how often and how much liquid and solid oats need to be consumed to reach quantifiable levels of AVAs in blood plasma. AVA content may vary within the same oat form and among different oat products. Therefore, we consider that the best approach of extrapolating the dose of AVAs (in nM) to dose of oat product (in g or mL) was to estimate an average content of AVAs from different commercial products, both from published data and measured values in our lab. The diagnostic plot suggests a good fit for the model, describing 75% of the variation of the observed data. The remaining unexplained variability may reflect inter-individual variability due to genetics, or other potential missing covariates from the model. In addition, the RMSE at 0.32 nM, reflects the sensitivity of the model to larger prediction errors, which is important to ensure effective dose predictions, and MAE (0.261 nM) indicates the average magnitude of prediction errors, providing an intuitive measure of typical model performance. Further, the predicted data are in good agreement with the observed data with an average underestimation of 0.145 nM, a value well within the limits of agreement (Fig. 6), indicating that the model does not exhibit systematic deviation from the experimental data. The prediction model was successfully utilized to estimate the conditions under which biomarkers of food intake reach quantifiable levels in blood circulation and a similar approach could be used to evaluate the utility of other food intake biomarkers. Our study included both sexes and a wide age span, to establish a general model for the pharmacokinetics of AVA and AVEs. However, given the relatively limited sample size and homogeneity in terms of ancestry and dietary culture, established parameters will need to be validated in larger and more diverse cohorts to confirm the robustness and generalizability of the model.

No previous studies, to our knowledge, have reported a simultaneous extraction of both prevalent AVAs (2p, 2c and 2f) and lesser known AVAs (2pd, 2fd). Moreover, this was the first study where AVAs and AVEs were quantified in blood plasma for the first time.

Although it is well known that other drug uses beyond antibiotics may affect gut microbiota composition and functionality, our inclusion of gut microbiota as potential determinants for AVA and AVE plasma concentrations were only exploratory, therefore we applied generous inclusion criteria related to drug use. Since we did not observe significant differences when testing AVAs to the relative abundance of *F. prausnitzii* it would be interesting to include the dihydro-metabolites in the same method for future studies and evaluate their presence in relation to gut microbiota. Exploratory analysis of associations between AVAs, AVEs, their metabolites and other gut microbiota species is also of our interest. Another strength of this study is the use of a compartmental model that allowed us to estimate doses and dose intervals and therefore evaluate the utility of AVAs and AVEs as biomarkers. The presented PK parameters and estimation can in future study designs be considered for dose and dosing interval of oats. The application, though, of this prediction is limited to the presented food matrices, as food processing and co-existing nutrients may affect the PK parameters and add further uncertainties to the estimation. The estimation may be expanded to other food matrices when data of similar study designs for other oat products are available.

## Conclusions

In conclusion, AVAs and AVEs may be of limited use for reflection of long-term habitual oat intake in epidemiological studies due to the short half-life and relatively low bioavailability, respectively. However, AVAs are good candidates as compliance biomarkers in oat intervention studies and their plasma levels should be investigated in relation to clinical outcomes to evaluate their potential bioactivities in humans.

## Supplementary Information

Supplementary Material 1.

Supplementary Material 2.

Supplementary Material 3.

Supplementary Material 4.

Supplementary Material 5.

Supplementary Material 6.

## Data Availability

No datasets were generated or analysed during the current study.

## Abbreviations

ADME: Absorption, Distribution, Metabolism, Excretion
AVA: Avenanthramide
AVE: Avenacoside
BMI: Body Mass Index
GI: Gastrointestinal
LC-MS/MS: Liquid chromatography-targeted mass spectrometry
MRM: Multiple reaction monitoring
PK: Pharmacokinetics
