# Supplementary material for: Avenanthramides and avenacosides as biomarkers of oat intake: a pharmacokinetic study of solid and liquid oat consumption under single and repeated dose conditions
Source: Nutr J. 2025 Sep 9;24:136. doi: 10.1186/s12937-025-01204-7 (PMC12418703; doi:10.1186/s12937-025-01204-7)
Supplement: Supplementary file 1 — Supplementary Material 1. [file 12937_2025_1204_MOESM1_ESM.docx]

| **AVA 2p** | | | | |
| --- | --- | --- | --- | --- |
| **Parameter (unit)** | **Liquid (RSE%)** | **IIV (CV%)** | **Solid (RSE%)** | **IIV (CV%)** |
| **ka (1/h)** | 2.8 (9) | 23 | 1.6 (10) | 17 |
| **V (L)** | 113 (16) | 62 | 220 (22) | 43 |
| **ke (1/h)** | 0.52 (10) | 28 | 0.44 (17) | 31 |
| **AVA 2c** | | | | |
| **ka (1/h)** | 5.9 (18) | 23 | 2.8 (10) | 17 |
| **V (L)** | 500 (19) | 80 | 720 (22) | 80 |
| **ke (1/h)** | 0.43 (17) | 21 | 0.31 (17) | 67 |
| **AVA 2pd** | | | | |
| **ka (1/h)** | 2.4 (9) | 13 | 1.5 (10) | 11 |
| **V (L)** | 130 (16) | 56 | 190 (22) | 29 |
| **ke (1/h)** | 0.30 (13) | 33 | 0.27 (17) | 59 |
| **AVA 2fd** | | | | |
| **ka (1/h)** | 3.6 (10) | 15 | 2.2 (10) | 23 |
| **V (L)** | 210 (13) | 42 | 320 (22) | 39 |
| **ke (1/h)** | 0.52 (14) | 31 | 0.42 (17) | 14 |
| **AVA 2f** | | | | |
| **ka (1/h)** | 4.8 (3) | 76 | 3.8 (26) | 19 |
| **V (L)** | 1600 (34) | 79 | 2600 (5) | 47 |
| **ke (1/h)** | 0.24 (11) | 80 | 0.24 (11) | 80 |
| **AVE A** | | | | |
| **ka (1/h)** | 1.4 (3) | 54 | 2.7 (8) | 53 |
| **V (L)** | 5900 (3) | 32 | 9700 (2) | 47 |
| **ke (1/h)** | 0.21 (10) | 33 | 0.22 (6) | 39 |
| **AVE B** | | | | |
| **ka (1/h)** | 0.41 (9) | 63 | 0.45 (22) | 80 |
| **V (L)** | 2200 (3) | 37 | 2600 (21) | 56 |
| **ke (1/h)** | 0.27 (3) | 80 | 0.19 (20) | 62 |

**Table S1:** Estimated values for the pharmacokinetic parameters derived from the compartmental model together with relative standard error (RSE%) to reflect the precision of the model and coefficient of variation to reflect biological or technical variances
