# Supplementary material for: Avenanthramides and avenacosides as biomarkers of oat intake: a pharmacokinetic study of solid and liquid oat consumption under single and repeated dose conditions
Source: Nutr J. 2025 Sep 9;24:136. doi: 10.1186/s12937-025-01204-7 (PMC12418703; doi:10.1186/s12937-025-01204-7)
Supplement: Supplementary file 2 — Supplementary Material 2. [file 12937_2025_1204_MOESM2_ESM.docx]

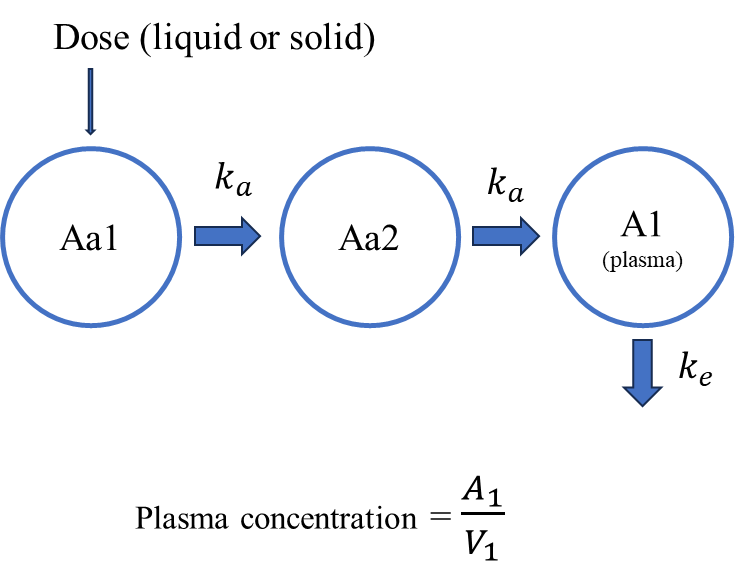


Figure S3: Visual description of the compartmental model, where Aa1 and Aa2 are the absorption compartments and A1 is the plasma compartment. ka, is the absorption rate, ke is the elimination rate and V1 is the distribution volume
