# Supplementary material for: Avenanthramides and avenacosides as biomarkers of oat intake: a pharmacokinetic study of solid and liquid oat consumption under single and repeated dose conditions
Source: Nutr J. 2025 Sep 9;24:136. doi: 10.1186/s12937-025-01204-7 (PMC12418703; doi:10.1186/s12937-025-01204-7)
Supplement: Supplementary file 3 — Supplementary Material 3. [file 12937_2025_1204_MOESM3_ESM.docx]

**Table S3:** Calculated LLODs and LLOQs of AVAs and AVEs.

| **Molecule** | **LLOD (nmol/L)** | **LLOQ (nmol/L)** |
| --- | --- | --- |
| **AVA 2p** | 0.20 | 0.67 |
| **AVA 2f** | 0.06 | 0.30 |
| **AVA 2c** | 0.16 | 0.32 |
| **AVE A** | 0.38 | 1.22 |
| **AVE B** | 0.35 | 1.06 |
