# Supplementary material for: Avenanthramides and avenacosides as biomarkers of oat intake: a pharmacokinetic study of solid and liquid oat consumption under single and repeated dose conditions
Source: Nutr J. 2025 Sep 9;24:136. doi: 10.1186/s12937-025-01204-7 (PMC12418703; doi:10.1186/s12937-025-01204-7)
Supplement: Supplementary file 4 — Supplementary Material 4. [file 12937_2025_1204_MOESM4_ESM.doc]

**
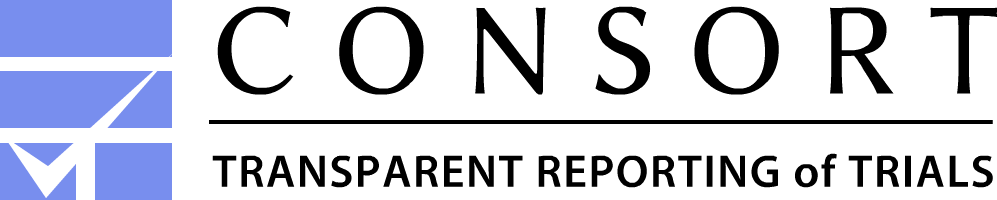
**

**CONSORT 2010 Flow Diagram**

**Allocation**

**Analysis**

**Follow-Up**

**Enrollment**

Assessed for eligibility (n= 38 )

Excluded (n= 15 )

♦  Not meeting inclusion criteria (n= 4 )

♦  Declined to participate (n=3 )

♦  Other reasons (n= 8 )

Analysed (n= 11 )
♦ Excluded from analysis (give reasons) (n= 0)

Lost to follow-up (give reasons) (n= 0 )

Discontinued intervention (give reasons) (n=0 )

Allocated to intervention (n=11 )

♦ Received allocated intervention (n= 11)

♦ Did not receive allocated intervention (give reasons) (n= 0)

Lost to follow-up (give reasons) (n=2 )

Discontinued intervention (n=1; difficulties with venous blood sampling, n=1;sickness on the second period) (total n=2 )

Allocated to intervention (n= 12)

♦ Received allocated intervention (n= 12)

♦ Did not receive allocated intervention (give reasons) (n= 0 )

Analysed (n=10 )
♦ Excluded from analysis (no samples available for analysis) (n= 2 )

Randomized (n= 23 )
