# Supplementary material for: Avenanthramides and avenacosides as biomarkers of oat intake: a pharmacokinetic study of solid and liquid oat consumption under single and repeated dose conditions
Source: Nutr J. 2025 Sep 9;24:136. doi: 10.1186/s12937-025-01204-7 (PMC12418703; doi:10.1186/s12937-025-01204-7)
Supplement: Supplementary file 5 — Supplementary Material 5. [file 12937_2025_1204_MOESM5_ESM.docx]

**Table S2:** Estimated median AVA content in liquid and solid oat product based on their reported content in the literature (for the solid product) and on experimental data from our laboratory (for the liquid product and for AVA 2pd and AVA 2fd). n corresponds to the number of products used for the estimations and a indicates that for the case of AVA 2pd and 2fd only experimental data from our lab were available

| **Content of AVAs in ug/g** | | | | | |
| --- | --- | --- | --- | --- | --- |
| **Product type** | **AVA 2p** | **AVA 2c** | **AVA 2f** | ***^a^*AVA 2pd** | ***^a^*AVA 2fd** |
| oat flakes (n=11) | 6.7 | 3.7 | 10.9 | 5.7 ^1^ | 9.0 ^1^ |
| oat milk (n=1) | 9.8 | 16.5 | 7.4 | 7.0 | 10.3 |

^1^ Median values for AVA 2pd and AVA 2fd in the solid product were estimated from experimental data of our laboratory.
