# Supplementary material for: Avenanthramides and avenacosides as biomarkers of oat intake: a pharmacokinetic study of solid and liquid oat consumption under single and repeated dose conditions
Source: Nutr J. 2025 Sep 9;24:136. doi: 10.1186/s12937-025-01204-7 (PMC12418703; doi:10.1186/s12937-025-01204-7)
Supplement: Supplementary file 6 — Supplementary Material 6. [file 12937_2025_1204_MOESM6_ESM.docx]

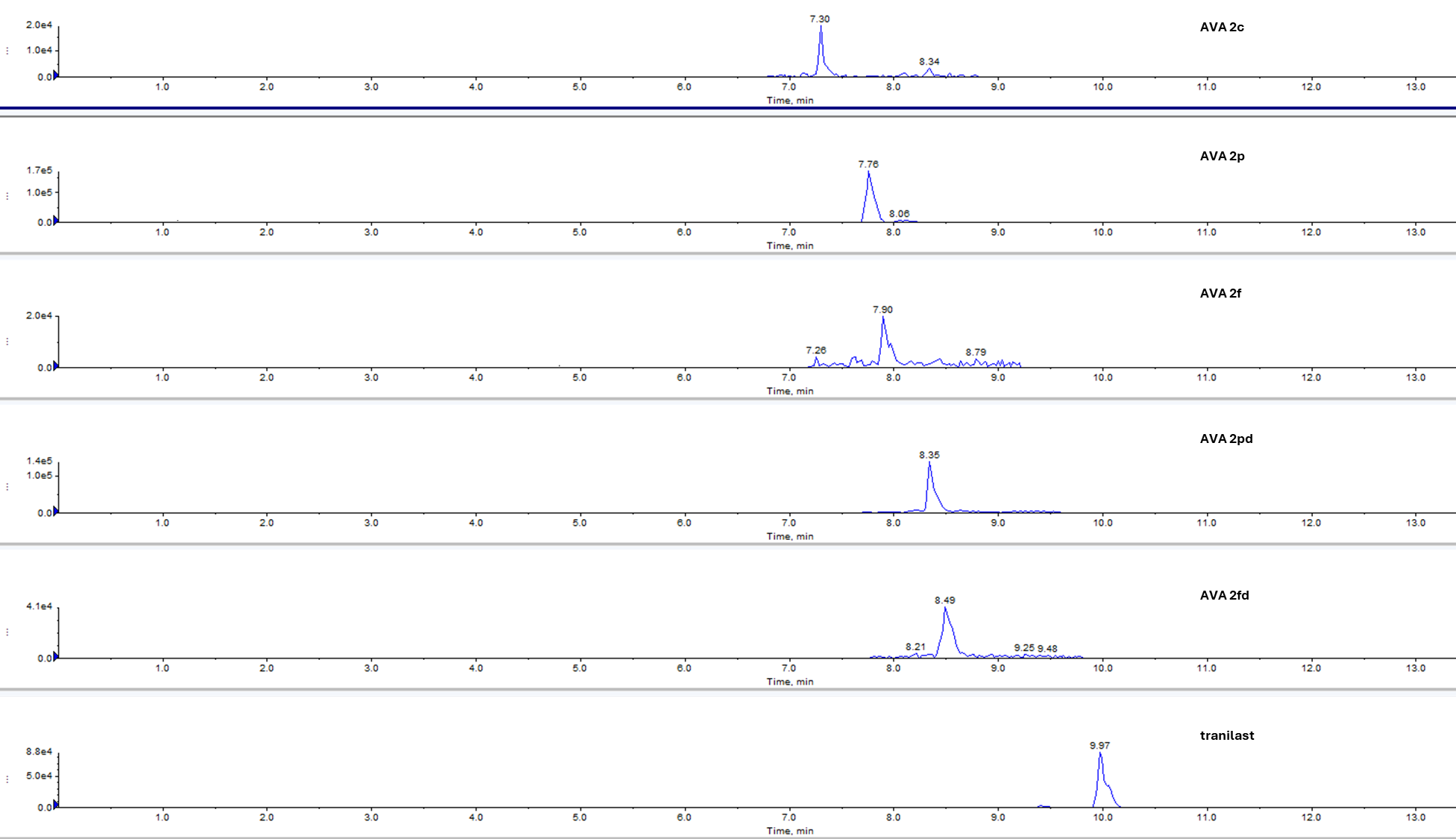


**A**

Figure S2: Typical chromatographic peaks and retention times of AVAs and AVEs as they were recorded in samples. Figure A, presents peak and elution sequence of AVAs and Tranilast^®^ in positive mode and figure B presents peak intensity and elution sequence of AVEs and Ginsenoside Rb1 in negative mode.


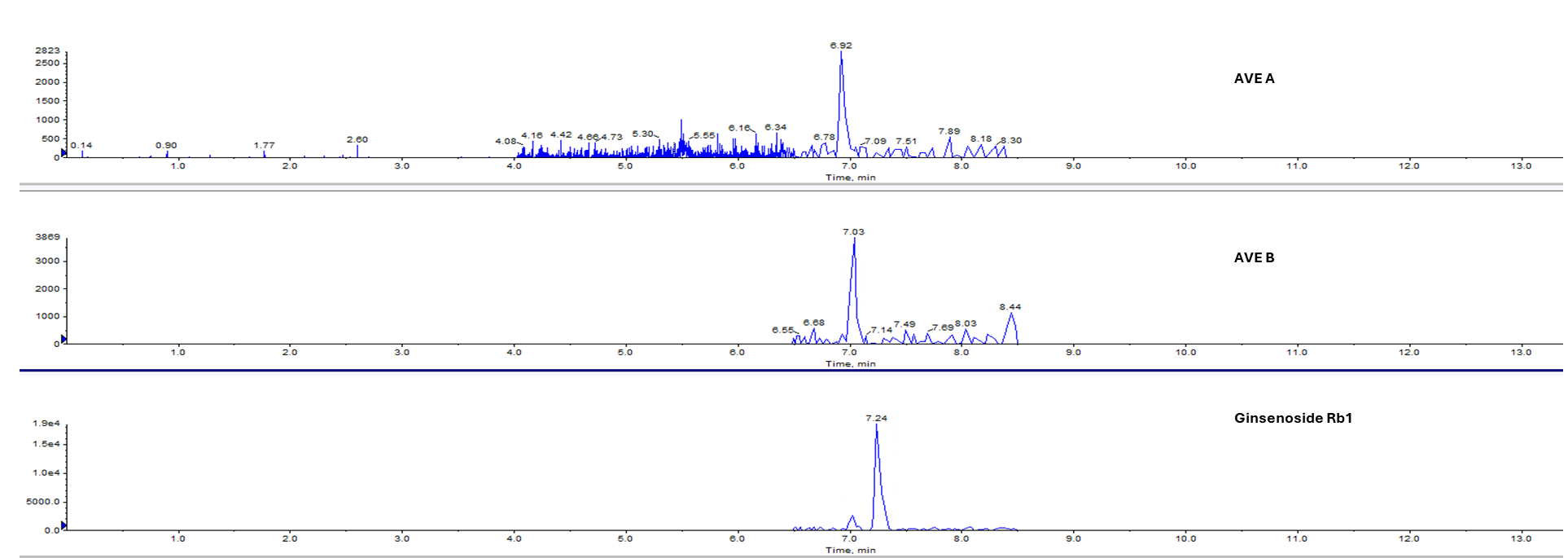


**B**

AVEs and AVAs respectively.
